# Bones and all: a new critically endangered Pantepui species of *Stefania* (Anura: Hemiphractidae) and a new osteological synapomorphy for the genus

**DOI:** 10.1186/s40851-023-00209-6

**Published:** 2023-05-25

**Authors:** Philippe J. R. Kok

**Affiliations:** 1 Department of Ecology and Vertebrate Zoology, Faculty of Biology and Environmental Protection, University of Łódź, 12/16 Banacha Str, 90-237 Łódź, Poland; 2 Department of Life Sciences, The Natural History Museum, Cromwell Road, London, SW7 5BD UK

**Keywords:** Endemism, Genetic distances, Homoplasy, Morphology, Osteology, µCT scanning, Symplesiomorphy, Systematics, Taxonomy

## Abstract

The hemiphractid frog genus *Stefania* is one of the many ancient (near-) endemic lineages of vertebrates inhabiting the biodiverse Pantepui biogeographical region in the Guiana Shield Highlands of northern South America—the famous “Lost World” of Arthur Conan Doyle. Previous molecular analyses of the genus *Stefania* have indicated that species boundaries and phylogenetic relationships are often incongruent with morphological traits in that clade. A substantial number of “taxonomically cryptic” species, often microendemic, remain to be described. This is notably the case for an isolated population from the summit of Wei-Assipu-tepui, a small table-top mountain at the border between Guyana and Brazil. That population was previously known as *Stefania* sp. 6 and belongs to the *S. riveroi* clade. The new species is phylogenetically distinct, but phenotypically extremely similar to *S. riveroi*, a taxon found only on the summit of Yuruaní-tepui in Venezuela and recovered as sister to all the other known species in the *S. riveroi* clade. The new taxon is described based on morphology and osteology. Data about genetic divergences within the *S. riveroi* clade are provided. A new synapomorphy for the genus *Stefania* is proposed: the presence of a distal process on the third metacarpal. Amended definitions are offered for the three other species in the *S. riveroi* clade (*S. ayangannae*, *S. coxi*, *S. riveroi*). The new species should be listed as Critically Endangered according to IUCN criteria.

**Supplementary Information:**

The online version contains supplementary material available at 10.1186/s40851-023-00209-6.

## Background

**Fig. 1:**
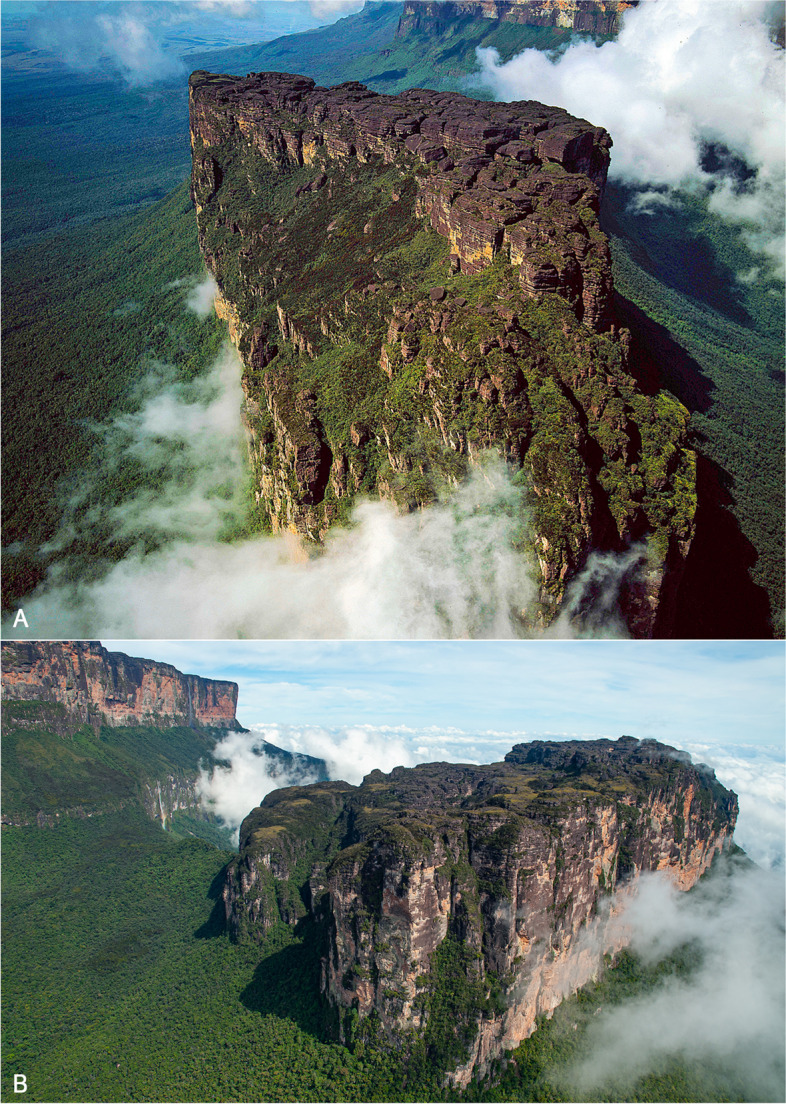
Summit of Wei-Assipu-tepui, terra typica of *Stefania maccullochi* **sp. nov. A** Aerial photograph taken facing southwest (photo Adrian Warren). **B** Aerial photograph taken facing northwest (photo by the author)

**Fig. 2:**
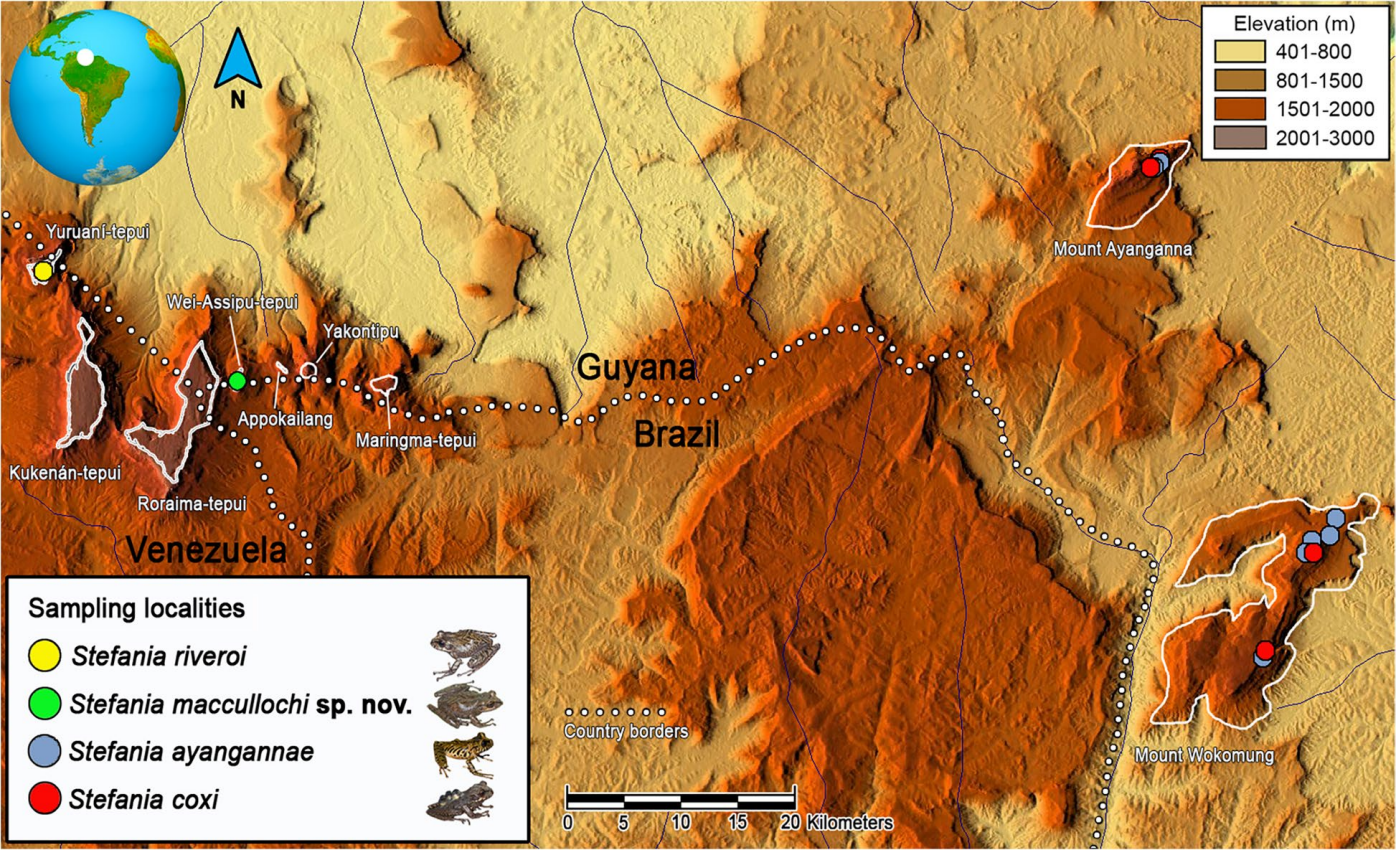
Distribution map of the *Stefania riveroi* clade as currently understood. Locality data are based on specimens examined (see Additional file 2: Appendix), literature records [7–13], and GBIF [14]. Type localities for *S. ayangannae* and *S. coxi* are Mount Ayanganna, Guyana. Inset photos by the author (*S. maccullochi* **sp. nov.** and *S. riveroi*) and D. B. Means (*S. ayangannae* and *S. coxi*)

Wei-Assipu-tepui is a poorly explored sandstone table-top mountain (tepui) located at the border between Guyana and Brazil and reaching a maximum elevation of ca. 2260 m (Figs. 1, 2). Its cliff-protected summit—only accessible by technical climb or helicopter—covers an area of about 3 km^2^ and is fractured by many large crevices, such as the *Sima de los Guácharos* reported to be more than 100 m deep [1] (Fig. 1). The first exploration of Wei-Assipu-tepui apparently dates back to July 2000 when the *Sociedad Espeleológica Italiana* and the *Sociedad Venezolana de Espeleología* led a joint helicopter expedition to its summit, with the aim to explore its cave system [1]. A small collection of amphibians was made at the time (6 species, 10 individuals), and an annotated list of its fauna was published [2]. The list included a new species of *Oreophrynella* (later described as *O. weiassipuensis* [3]), and an unidentified *Stefania* species. I spent more than 2 weeks on Wei-Assipu-tepui in November 2009, which resulted in the collection of 9 amphibian and reptile taxa (some new, e.g., [4]), including 23 specimens of a *Stefania* species phenotypically undistinguishable from *Stefania riveroi* from Yuruaní-tepui in Venezuela. However, a few years later Kok et al. [5, 6], based on a multilocus DNA phylogeny, demonstrated that the population from Wei-Assipu-tepui and *Stefania riveroi* are not reciprocally monophyletic*.* Kok et al. [6] treated this undescribed taxon as *Stefania* sp. 6. Both *Stefania riveroi* and the morphologically cryptic *S.* sp. 6 are members of what Kok et al. [6] named the *S. riveroi* clade, which also comprises *S. coxi* and *S. ayangannae*, the latter recovered sister to *S.* sp. 6 [6]*.* The purpose of this paper is to formally describe and name this new species, which is seemingly endemic to the small summit of Wei-Assipu-tepui and can only be distinguished from *S. riveroi* by DNA and a few subtle osteological characters. Based on new data and comparisons, I also propose new amended definitions for the 3 other species in the *S. riveroi* clade, and a new synapomorphy for the genus: the presence of a distal process on the third metacarpal.

## Methods

### Field work and deposition of specimens

Specimens were collected by hand and sacrificed by immersion in 2% Xylocaine. Tissue samples (a piece of liver and/or thigh muscle) were removed from most specimens and preserved in absolute ethanol. Whole individuals were fixed in 10% formalin and later transferred to 70% ethanol for permanent storage. Specimens have been deposited in the collections of the Natural History Museum (NHMUK; London, United Kingdom), the Royal Ontario Museum (ROM; Toronto, Canada) and the Royal Belgian Institute of Natural Sciences (IRSNB; Brussels, Belgium).

### Morphometrics and morphological data

Morphological examinations and measurements of the type series were performed under a Leica M205C stereomicroscope. Morphometric data were taken from the preserved specimens to the nearest 0.01 mm (rounded to 0.1 mm) with digital callipers (MarCal 16 EWRi). Morphological data from the referred specimens were retrieved from Russo [15]. Morphological comparisons are based on examination of museum specimens (see Additional file 2:  Appendix) and published descriptions [7–11, 16–23]. Description of external morphological characters follows Kok and Kalamandeen [24]. Definition, diagnosis, and description of the holotype mostly follow the scheme of MacCulloch and Lathrop [8], with amendments as provided in Kok [25], for ease of comparison.

### µCT scanning, 3D reconstructions, and osteology

The holotype was µCT-scanned at the NHMUK’s CT Lab facility using a Nikon HMX225; osteological images were exported from the virtual 3D models, which were reconstructed and segmented using VGStudio MAX version 2.1. Comparative specimens of *S. ayangannae*, *S. coxi*, and *S. riveroi* have been µCT-scanned, reconstructed, and segmented (using Dragonfly) by J. Brecko at the IRSNB’s CT Lab facility using an RX EasyTom150. Full-body three-dimensional mesh files have been deposited either on the MorphoSource (holotype) or on the Sketchfab platform (comparative specimens) (Additional file 1: Table S1). Osteological terminology followed Trueb [26] and Duellman [27]. The degree of contact between bony structures followed Kok et al. [28], i.e., contacting/in contact = contact between structures with a visible suture line, and fused = contact between structures with a suture line being barely visible or absent.

#### Genetic distances

Uncorrected pairwise genetic distances among species within the *S. riveroi* clade were calculated in MEGA X [29], using 16S sequences deposited by Kok et al. [6] and available on GenBank. Sequences were aligned using MAFFT v7.490 [30] on the CIPRES Science Gateway [31] with appropriate strategies automatically selected. Ambiguously aligned regions were manually removed, resulting in a final alignment of 511 nucleotides.

## Results

*Stefania maccullochi* **sp. nov. **https://zoobank.org/urn:lsid:zoobank.org:pub:959E71C1-8E5A-4CA1-90E8-8D8E8C8F01EB

*Stefania* sp. Villarreal et al. 2002 [2]: 48.

*Stefania* sp “Wei-Assipu” Kok et al. 2012 [5]: Supplemental Information.

*Stefania* sp. 6 Kok et al. 2017 [6]: 175–176.

**Fig. 3:**
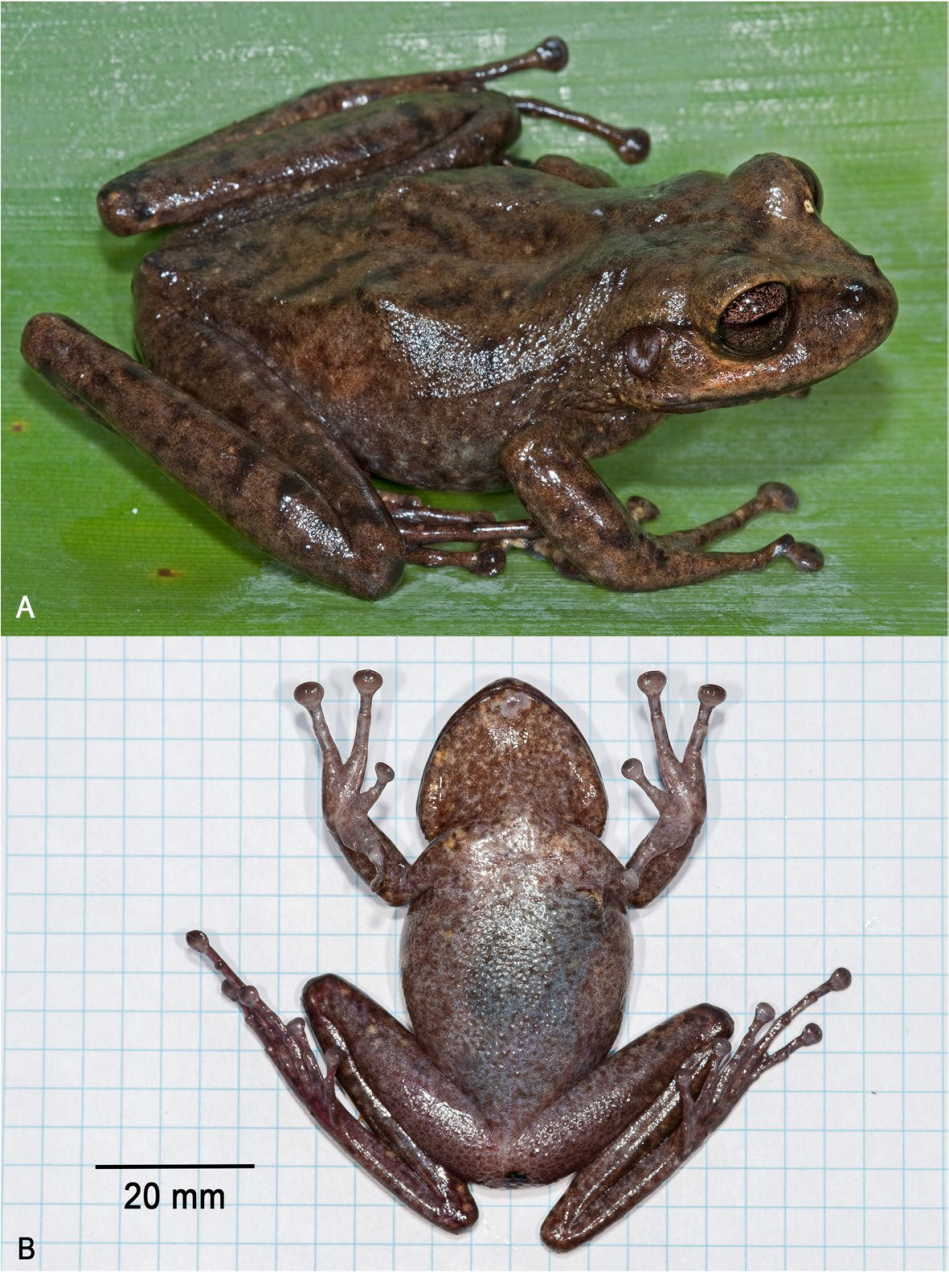
Holotype of *Stefania maccullochi* **sp. nov. **(NHMUK 2023.3184, female, 62.9 mm SVL) **A** Dorsolateral view in life. **B** Ventral view of the specimen freshly euthanized. Photos by the author

**Fig. 4:**
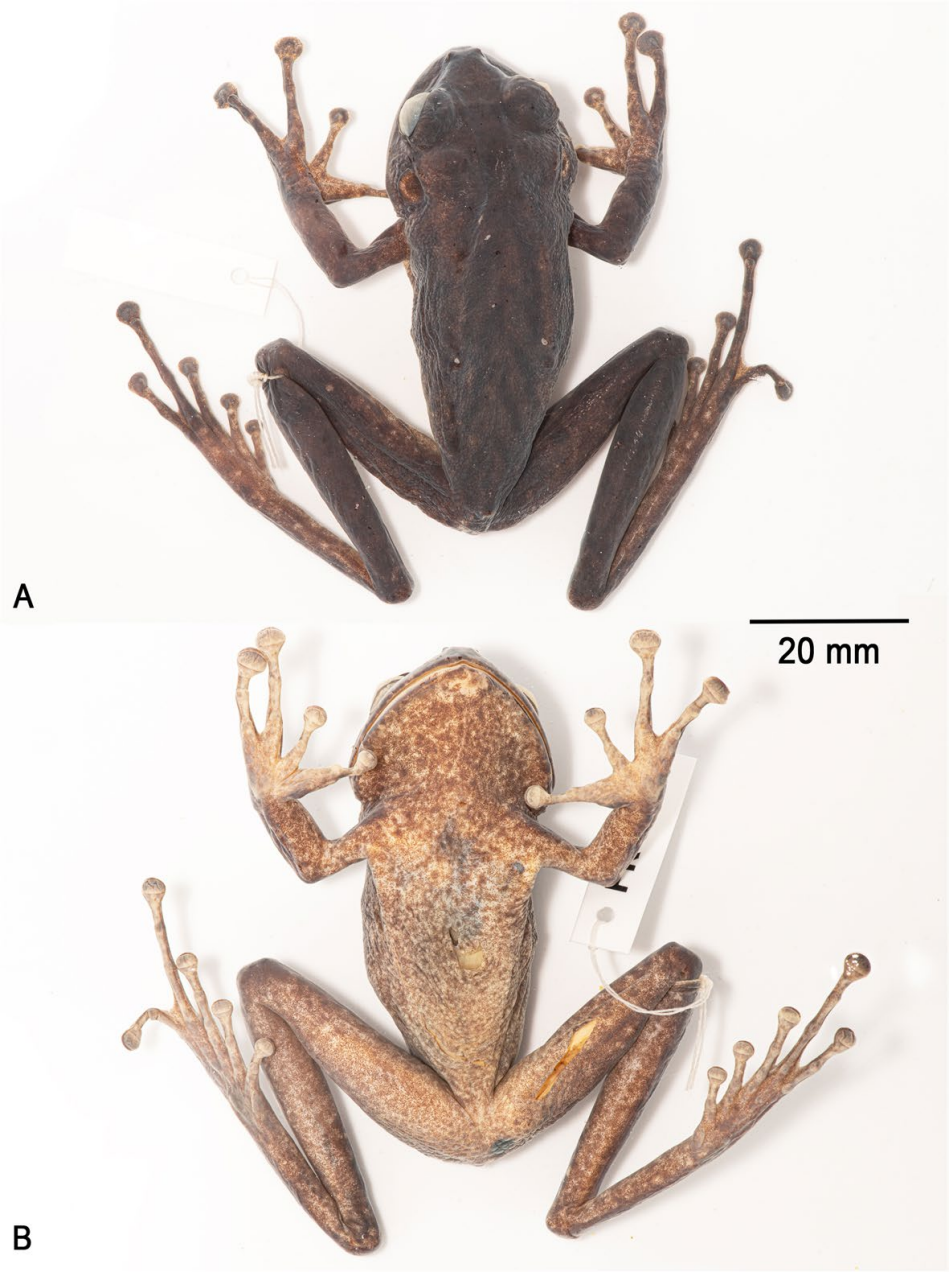
Holotype of *Stefania maccullochi* **sp. nov.** (NHMUK 2023.3184, female, 62.9 mm SVL) in ethanol preservative **A**. Dorsal view. **B**. Ventral view. Photos by the author

**Fig. 5:**
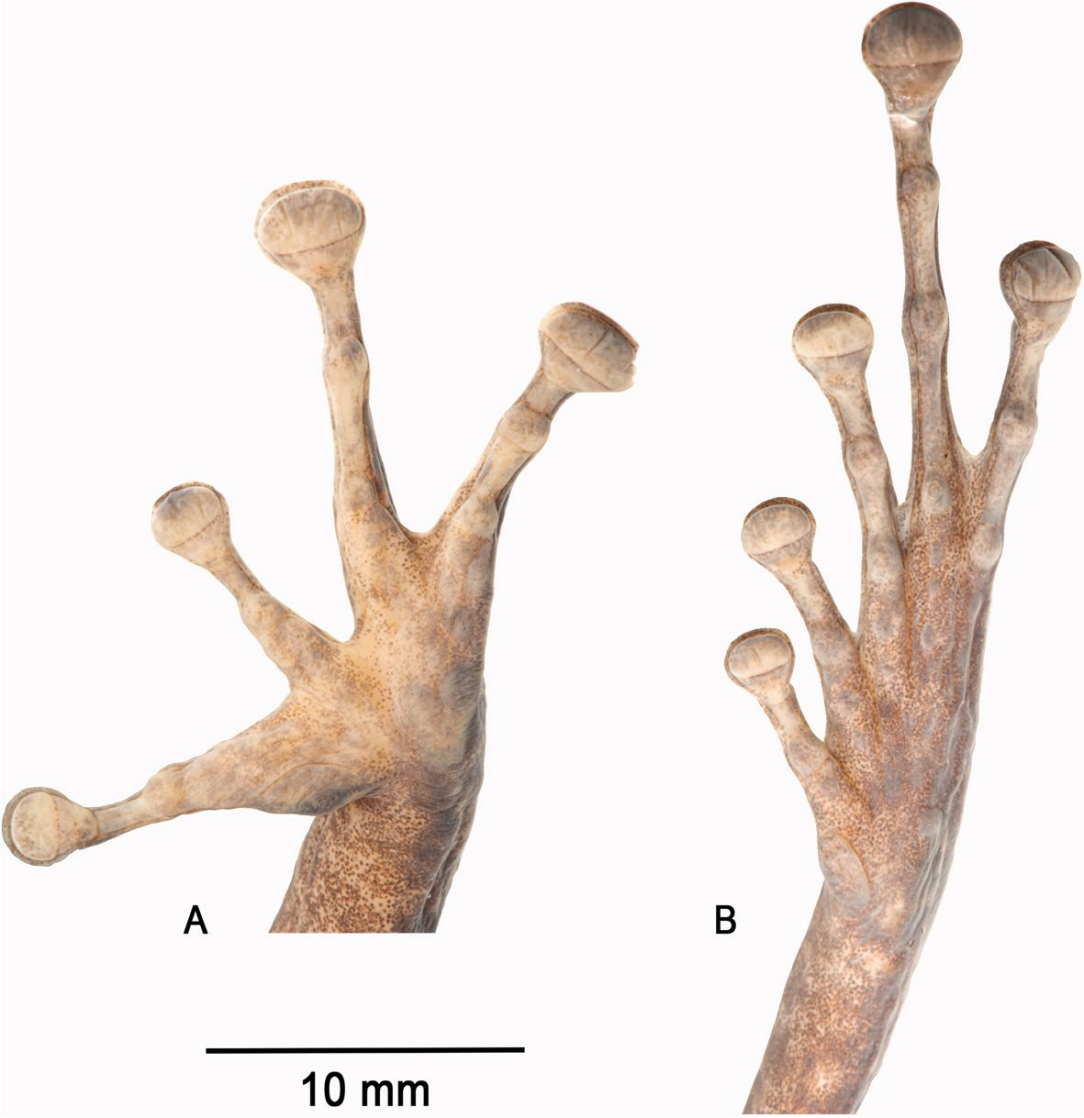
Palm (**A**) and sole (**B**) of the preserved holotype of *Stefania maccullochi* **sp. nov.** (NHMUK 2023.3184). Photos by the author

**Fig. 6:**
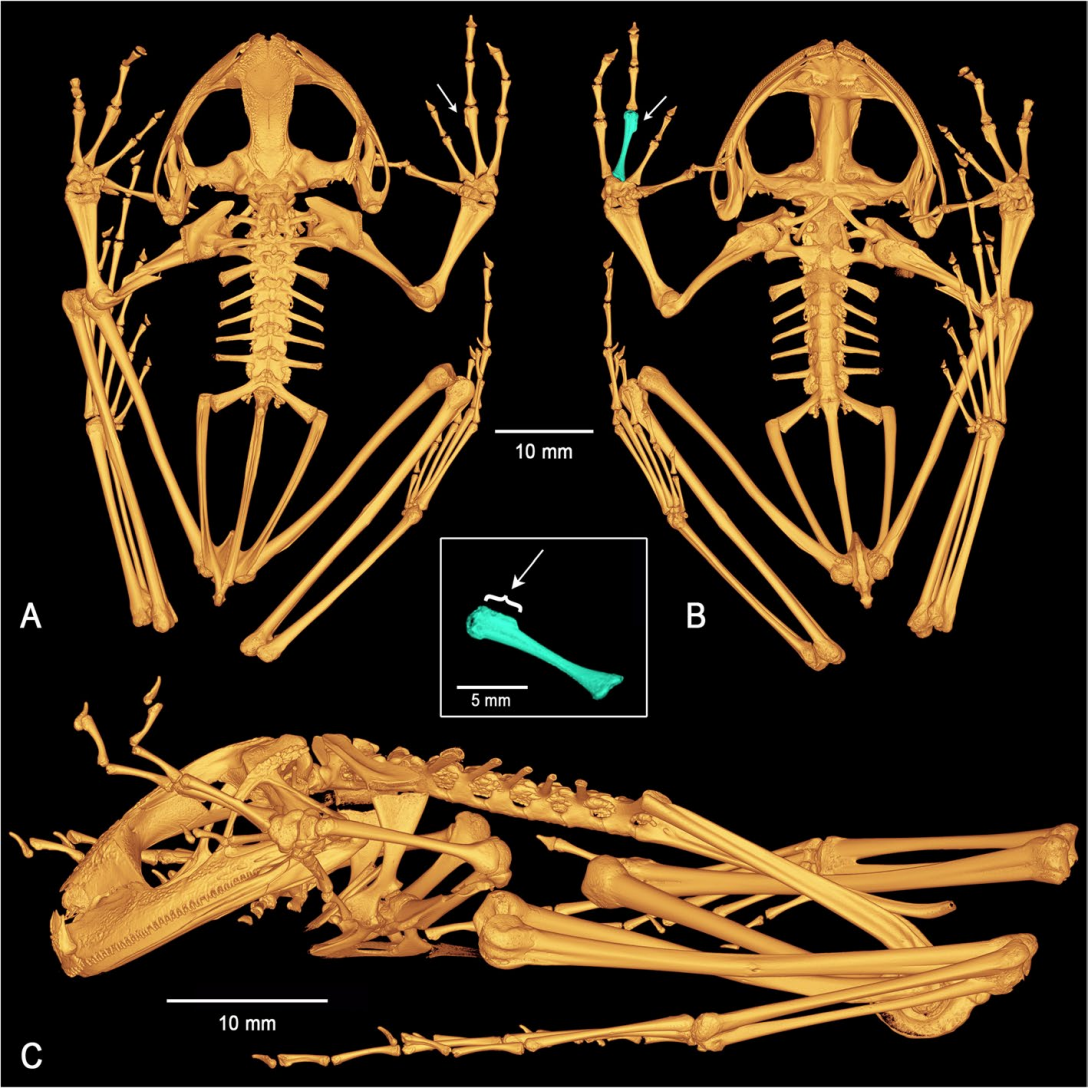
Three-dimensional model of the complete skeleton of the holotype of *Stefania maccullochi* **sp. nov. **(NHMUK 2023.3184) based on µCT imagery. **A** Dorsal view. **B** Ventral view. **C** Left lateral view. White arrows indicate the presence of a distal process on the third metacarpal, a new synapomorphy for the genus. Central inset highlights the third metacarpal of the right hand of the holotype in ventral view and shows the distal process

**Fig. 7:**
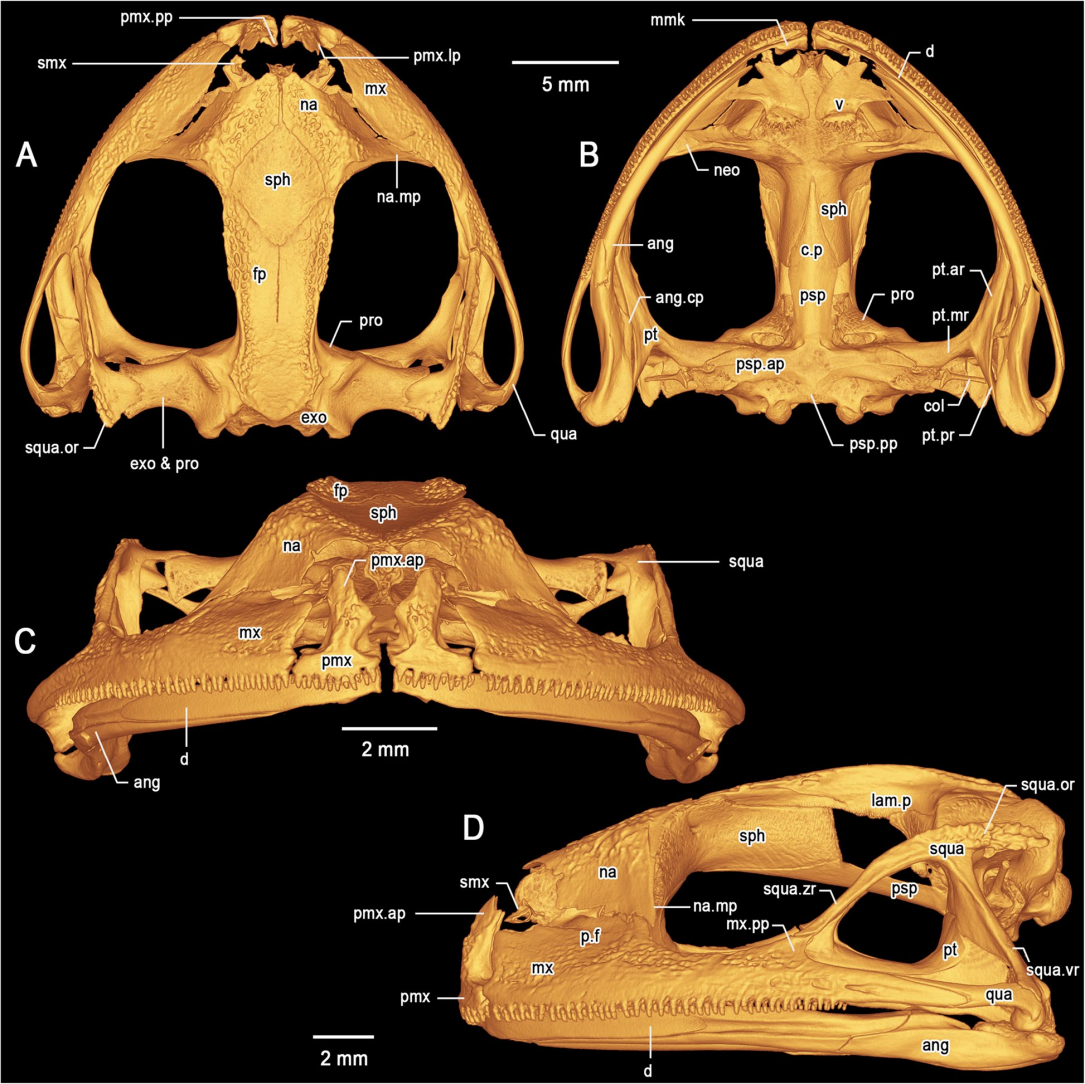
Three-dimensional model of the cranium of the holotype of *Stefania maccullochi* **sp. nov.** (NHMUK 2023.3184) based on µCT imagery. **A** Dorsal view. **B** Ventral view. **C** Frontal view. **D** Left lateral view. Abbreviations: *ang* Angulosplenial, *ang.cp* Coronoid process of the angulosplenial, *col* Columella, *c.p *Cultriform process, *d* Dentary, *exo* Exoccipital, *fp* Frontoparietal, *lam.p* Lamina perpendicularis, *mmk* Mentomeckelian, *mx *Maxilla, *mx.pp* Posterodorsal projection of the maxilla, *na* Nasal, *na.mp* Maxillary process of the nasal, *neo* Neopalatine, *p.f  *Pars facialis, *pmx* Premaxilla, *pmx.ap* Alary process of the premaxilla, *pmx.lp* Lateral process of the premaxilla, *pmx.pp* Palatine process of the premaxilla, *pro* Prootic, *psp* Parasphenoid, *psp.ar* Alary process of the parasphenoid, *psp.pp* Posteromedial process of the parasphenoid, *pt *Pterygoid, *pt.ar* Anterior ramus of the pterygoid, *pt.mr* Medial ramus of the pterygoid, *pt.pr* Posterior ramus of the pterygoid, *qua* Quadratojugal, *smx* Septomaxilla, *sph* Sphenethmoid, *squa* Squamosal, *squa.or* Otic ramus of the squamosal, *squa.vr* Ventral ramus of the squamosal, *squa.zr* Zygomatic ramus of the squamosal, *v* Vomer

**Holotype**. NHMUK 2023.3184 (field number PK2071, Figs. 3, 4, 5, 6, 7), an adult female collected by Philippe J. R. Kok, Paul Benjamin, and Claudius Perry, 3 November 2009 at 19h30, summit of Wei-Assipu-tepui, Cuyuni-Mazaruni, Guyana (05°13′05″N, 060°42′15″W; 2210 m elevation).

**Paratopotypes** (*n* = 17). ROM 60548 (subadult male; field number PK2060), NHMUK 2023.3185 (female with two near-term juveniles; field number PK2063), ROM 60549 (male; field number PK2076), ROM 60550 (male; field number PK2079), ROM 60551 (male; field number PK2080), ROM 60552 (male; field number PK2081), NHMUK 2023.3186 (male; field number PK2083), NHMUK 2023.3187 (male; field number PK2084), NHMUK 2023.3188 (female; field number PK2098), NHMUK 2023.3189 (female with nine juveniles; field number PK2099), NHMUK 2023.3190 (female with 10 eggs/metamorphs; field number PK2122), NHMUK 2023.3191 (male; field number PK2128), NHMUK 2023.3192 (male; field number PK2137), ROM 60553 (male; field number PK2138), NHMUK 2023.3193 (male; field number PK2148), NHMUK 2023.3194 (male; field number PK2159), ROM 60554 (juvenile; field number PK2142), all collected between 3–17/11/2009 by Philippe J. R. Kok, Paul Benjamin, and Claudius Perry.

**Referred specimens** *(n* = 5). IRSNB 15853 (female with four near-term juveniles; field number PK2064), IRSNB 15854 (male; field number PK2096), IRSNB 15855 (female; field number PK2147), IRSNB 18705 (female; field number PK2129), IRSNB 18706 (female; field number PK2135), all collected between 3–17/11/2009 by Philippe J. R. Kok, Paul Benjamin, and Claudius Perry on the summit of Wei-Assipu-tepui, Cuyuni-Mazaruni District, Guyana.

### Etymology

The specific epithet *maccullochi* is a noun in the genitive case, honoring Canadian herpetologist Ross Douglas MacCulloch (born 1948) for his seminal contribution to the systematics and taxonomy of the genus *Stefania* in particular, and to the knowledge of the amphibians and reptiles of Guyana in general.

### Definition and diagnosis

*Stefania maccullochi* **sp. nov.** is characterized by the combination of the following morphological characters that distinguish it from all known congeners: (1) a large species of *Stefania*, max SVL in preserved females 72.9 mm, 54.6 mm in preserved males; (2) head not distinctly longer than wide, about as wide as long; (3) canthus rostralis smooth, prominent, rounded, concave, canthal stripe present in life, sometimes inconspicuous, rarely broad; (4) loreal region with a few low tubercles; (5) upper eyelid mostly smooth, absence of enlarged triangular appendage on its posterior upper part, although a conical tubercle may be present medially; (6) frontoparietal ridges conspicuous, low (in life/preservative); (7) frontoparietal crests present, feebly developed, laterally projecting (on cranium); (8) constriction of the frontoparietal bones at the level of the anterior epiotic eminence; (9) extensive, albeit low, exostosis on the cranium; (10) premaxillae inclined posteriorly; (11) posterodorsal projection of maxilla in contact with orbital/zygomatic ramus of squamosal; (12) maxillary process of the nasal barely in contact with the maxilla; (13) horizontal length of tympanum more than 50% horizontal length of eye in both sexes; (14) vomerine teeth 3–9; (15) Toes II–V basally webbed, no significant difference in toe webbing between sexes; (16) dorsal skin (in life) shagreened, with or without a few sparse enlarged tubercles; (17) ventral skin (in life) granular; (18) absence of conspicuous outer tarsal tubercles (in life); (19) absence of multiple conspicuous dark brown bars on flanks and lips, absence of dorsolateral stripes (in life); (20) in living adults, iris unicolor, golden yellow to copper, with extensive dark brown reticulations.

**Fig. 8:**
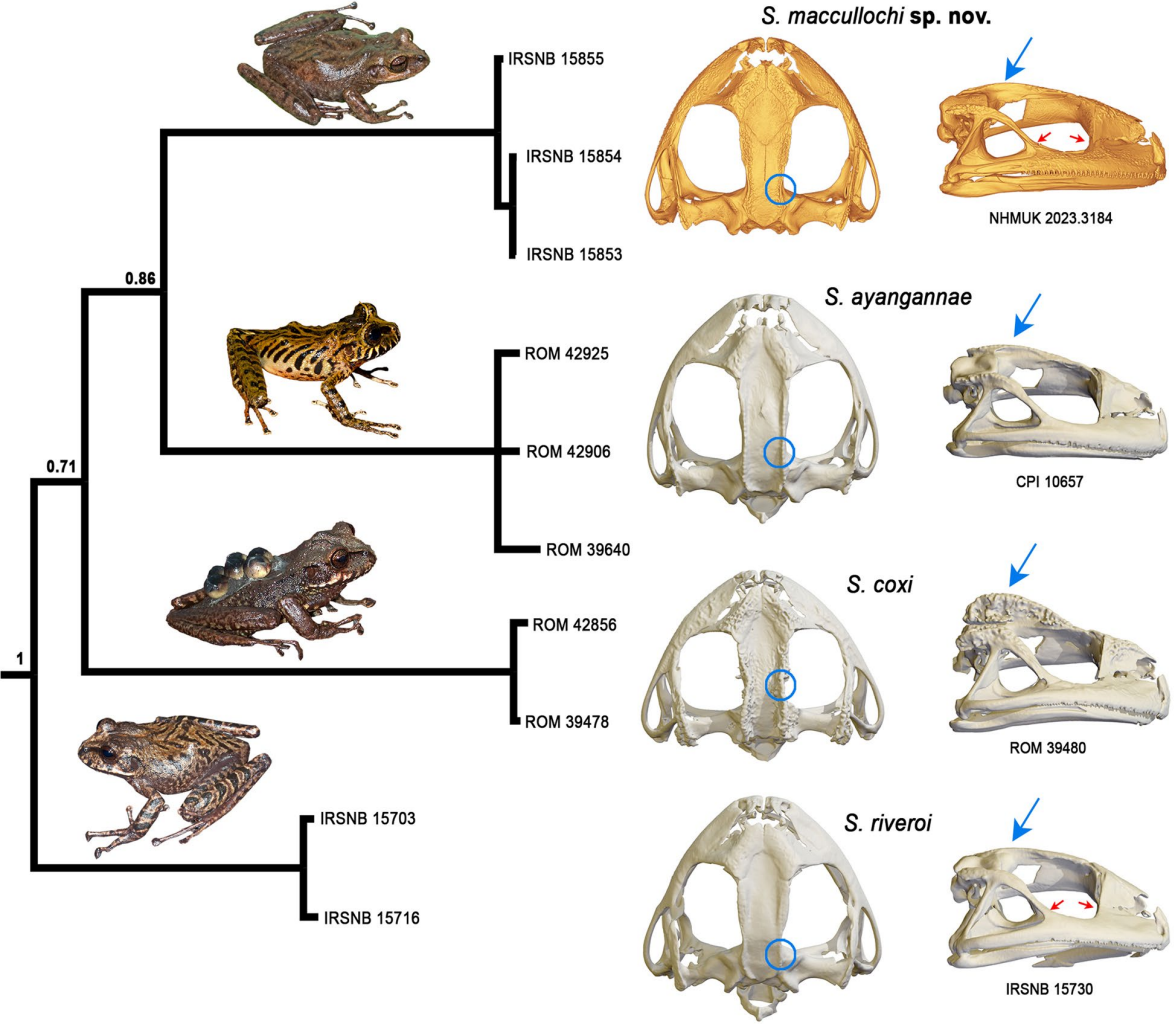
Phylogenetic relationships of the *Stefania riveroi* clade modified from [6], based on 2301 base pairs of nuclear and mitochondrial DNA (Bayesian statistical supports are provided at nodes), and comparison of crania in dorsal and lateral views. Circles and arrows highlight potential diagnostic characters: blue circles highlight absence/presence/location of a constriction in the frontoparietal bones; blue arrows highlight the condition of the frontoparietal crests; red arrows highlight the condition of (1) the contact between the posterodorsal projection of the maxilla and the orbital/zygomatic ramus of the squamosal, and (2) the contact between the maxillary process of the nasal and the maxilla. Inset photos by the author (*S. maccullochi* **sp. nov. **and *S. riveroi*) and D. B. Means (*S. ayangannae* and *S. coxi*)

**Fig. 9:**
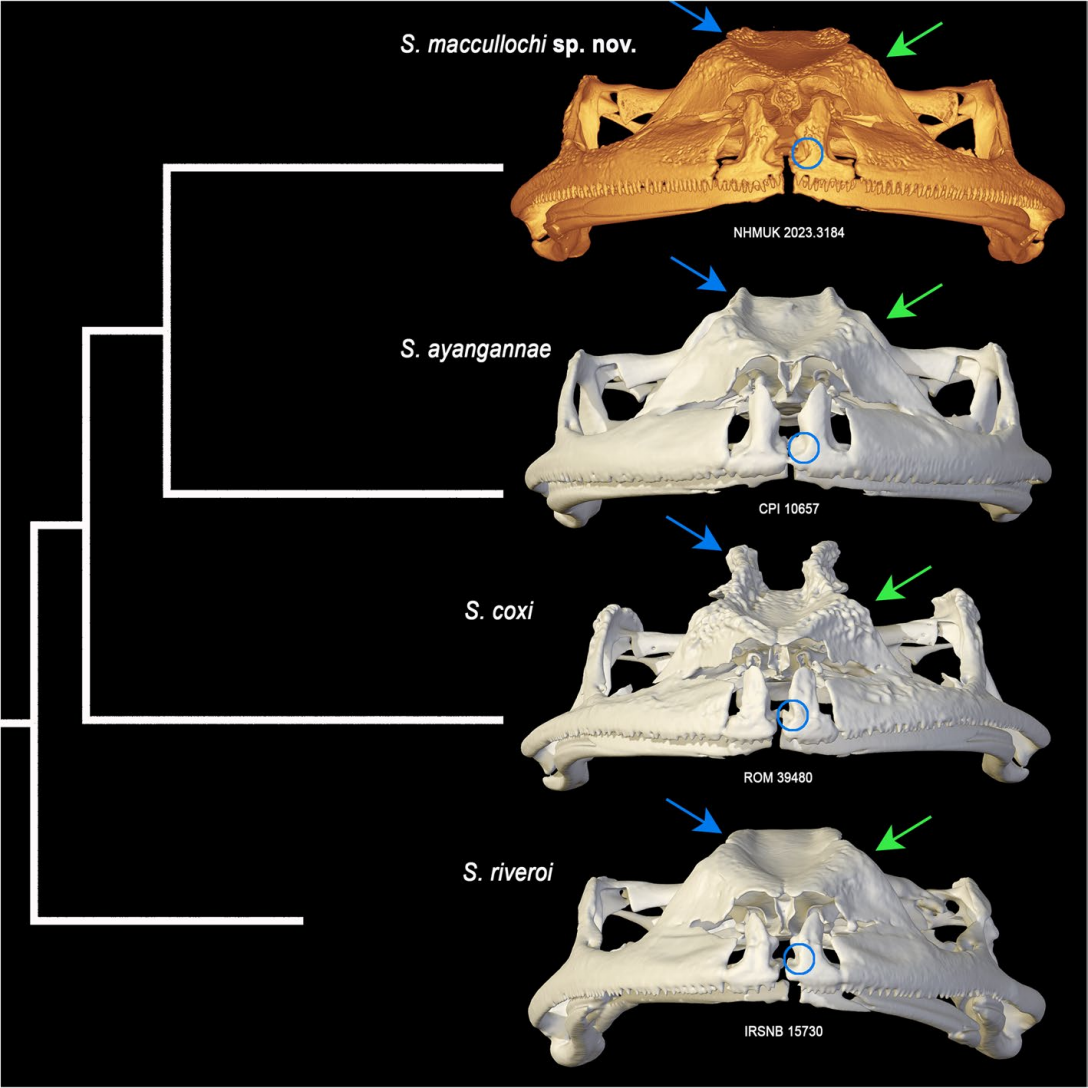
Phylogenetic relationships of the *Stefania riveroi* clade (modified from [6]), and comparison of crania in frontal view. Circles and arrows highlight diagnostic and potentially diagnostic characters: blue arrows highlight the condition of the frontoparietal crests; green arrows highlight the condition of exostosis; blue circles highlight the condition/absence of an indentation along the anteromedial base of the alary process of the premaxilla

More specifically, *Stefania maccullochi* **sp. nov.** may be distinguished from other members of the *S. riveroi* clade as follows:

From *Stefania ayangannae* (sister species according to Kok et al. [6]) notably by a much larger SVL (max 72.9 mm vs max 54.8 mm in *S. ayangannae*); the absence of conspicuous outer tarsal tubercles in life (present in *S. ayangannae*); the absence of multiple conspicuous dark brown bars on flanks and lips (present in *S. ayangannae* [8, 11]; Fig. 8); the presence of extensive, albeit low, exostosis (dermal sculpturing) on the cranium (less exostosed in *S. ayangannae*, except on the frontoparietal crests; Figs. 8, 9); poorly developed, laterally projecting, frontoparietal crests (moderately developed and projecting dorsally in *S. ayangannae*; Figs. 8, 9); and constriction of the frontoparietals at the level of the anterior epiotic eminence (absence of constriction in *S. ayangannae*; Fig. 8).

From *Stefania coxi* notably by the presence of low exostosis (dermal sculpturing) on the cranium (highly exostosed in *S. coxi*; Figs. 8, 9); poorly developed frontoparietal crests (highly developed in *S. coxi*; Figs. 8, 9); the absence of conspicuous outer tarsal tubercles in life (present in *S. coxi*); and constriction of the frontoparietals at the level of the anterior epiotic eminence (constriction anterior to the anterior epiotic eminence in *S. coxi*; Fig. 8).

*Stefania maccullochi* **sp. nov.** and *S. riveroi* are both large species reaching ca. 70 mm SVL with similar skin texture in life and high intraspecific polychromatism, which makes them difficult—if not impossible—to differentiate based on external morphology alone, especially in preservative. To my knowledge, in addition to DNA, these two species can only be differentiated by subtle osteological characters (see Discussion): the presence of extensive, albeit low, exostosis (dermal sculpturing) on the cranium in *S. maccullochi* **sp. nov.** (slightly less exostosed in *S. riveroi*; Figs. 8, 9), a deep indentation along the anteromedial base of the alary process of the premaxilla in *S. maccullochi* **sp. nov.** (absent in *S. riveroi*; Fig. 9), the posterodorsal projection of the maxilla barely contacting the orbital/zygomatic ramus of squamosal (fused in *S. riveroi*; Fig. 8); the maxillary process of the nasal barely in contact with the maxilla (fused in *S. riveroi*; Fig. 8); and a slightly more developed frontoparietal crest in *S. maccullochi* **sp. nov.** (visible on the cranium, e. g., Figs. 8, 9, but poorly evident in life).

### Description of the holotype

An adult female 62.9 mm SVL (Figs. 3, 4, 5), in good condition except for incisions made during tissue sampling (left thigh, lower thorax) and during examination (ventral V-shaped dissection to access internal organs). Head slightly longer than wide, distinctly wider than neck. Snout rounded in dorsal and lateral views, slightly longer than horizontal length of eye. Eye-nostril distance almost 3/4 of horizontal length of eye, more than three times the distance between the nostril and the tip of snout. Canthus rostralis prominent, rounded, concave; loreal region concave, sloping; lips flared. Nostrils protuberant, directed anterolaterally. Internarial distance ca. 60% of interorbital distance, 2.5 times distance between nostril and tip of snout. Internarial region concave. Interorbital space approximately equal to upper eyelid width. Frontoparietal ridges conspicuous, low. Temporal region bulged. Tympanum distinct, large, round, directed posterolaterally, 64% of horizontal length of eye, separated from eye by ca. 38% of horizontal length of eye. Supratympanic fold prominent, extending from posterior corner of eye to above insertion of forelimb, obscuring upper margin of tympanum.

Choanae large, oval. Vomerine processes transverse between choanae, not in contact, larger than choanae, each bearing ca. nine teeth. Tongue large, round. Palpebral membrane not reticulated, upper rim with a light brown band.

Dorsal skin shagreened; venter and posterior proximal surface of thighs granular. Upper eyelid smooth, with a small medial conical tubercle. Loreal region with a few inconspicuous tubercles. Tympanic region with a few small tubercles. Canthus rostralis smooth. Cloacal opening directed posteriorly at upper level of thighs, presence of a short cloacal flap.

Thenar tubercle large, distinct, projecting posteriorly, transversely oval; palmar tubercle large, distinct, bifid (somewhat heart-shaped; Fig. 5). Subarticular tubercles distinct, large, projecting, and single in appearance, although distal subarticular tubercles on FIII and FIV appear to be pairs of fused tubercles (Fig. 5). Supernumerary tubercles small and flat. Relative finger lengths II < I < IV < III; adpressed second finger reaches mid-length of the first finger’s disc. Fingers with lateral fringes; narrow webbing between FIII–FIV, which are fused proximally. Finger discs large, transversely oval, more than 2 times wider than adjacent phalange, smallest and equal in length on FI and FII, largest and equal in length on FIII–IV. Largest disc width 75% of horizontal length of tympanum.

Inner metatarsal tubercle large, oval, distinct; outer metatarsal tubercle 3 times smaller than inner, round, distinct (Fig. 5). Subarticular tubercles single, round, distinct. Supernumerary tubercles round, distinct. Relative lengths of toes I < II < III < V < IV; adpressed fifth toe slightly longer than third. Toe webbing absent between TI–II, other toes basally webbed, webs tapering to lateral fringes, fringes also present on outer margins of first and fifth toes, albeit much reduced in size on outer margin of the latter. Webbing formula I 3—3 II 2—3 ½ III 2^+^—4^−^ IV 3 ½—2^+^ V. Toe discs oval, wider than adjacent phalange, largest toe disc on Toe IV, subequal to largest finger disc. Heels overlapping when hindlimbs are folded at right angles to sagittal body plane.

### Color of the holotype in life

Dorsum medium brown with 4–5 faint dark brown chevrons and a few scattered dark brown marks (Fig. 3). Flanks medium brown with faint mottling. Anterodorsal aspect of upper and lower limbs medium brown with faint dark brown crossbands. Hands and feet medium brown with faint mottling and a few yellowish-brown spots. Canthal stripe present, inconspicuous, a narrow line; dark brown supratympanic stripe present; dark brown interorbital bar present. Central and upper portion of tympanum medium brown, surrounded by light grey. Upper lip creamy brown unmarked except for some scattered melanophores; lower lip dark brown with a few small creamy brown spots. Throat medium brown with fine light grey and yellowish-brown mottling; a larger light grey patch on chin. Venter and underside of limbs similar to throat, but with less dense mottling; presence of a few yellowish-brown spots on thorax and underside of limbs. Palms grey, suffused with melanophores, soles dark grey, with a red wash on the right sole. Iris unicolor, copper, with extensive dark brown reticulations.

### Color of the holotype in preservative

After 13 years in 70% ethanol preservative (Fig. 4), the overall coloration appears similar to the condition in life, except that all grey (light and dark) and yellowish-brown areas turned cream, making the mottling on the ventral face much more contrasted. Dark brown dorsal marks are essentially the same.

### Osteology of the holotype (Figs. 6, 7, 8, 9)

### Cranium (Figs. 7, 8, 9)

The skull is massive, mostly exostosed (covered with bony growths), widest at the level of the articulation of the quadratojugal and maxilla and wider than long (longest width ca. 120% of medial length). The braincase is well ossified; the sphenethmoid complex is mostly ossified, dorsally invested by the nasals along the entire anterior margin with suture lines visible. The prootic is overlapped laterally by the otic ramus of the squamosal and is in medial contact with the frontoparietal. The otic ramus of the squamosal and the prootic are contacting, but not fused. The paired septomaxillae are well developed and lie dorsal to the palatine process of the maxilla and posterolaterally to the articulation between the maxilla and premaxilla. The columellae (stapes) are well ossified, formed by the synostotic fusion of the long, thin pars media plectri (stylus) and the pars interna plectri (baseplate), which is curved.

#### *Dorsal investing bones* (Figs. 7, 8, 9)

The nasals are broad, exostosed, not contacting medially. The posteromedial margins of the nasals are in contact with the sphenethmoid, which projects anteriorly in front of the anterior border of the nasals. The maxillary process of the nasal is large, acuminate, barely in contact with the maxilla. Lateral margins of the frontoparietal are exostosed. The frontoparietal completely roofs the central braincase from the anterior level of the orbit to the level of the tectum synoticum posteriorly. Lamina perpendicularis poorly developed along the anterior orbital margin of the frontoparietal, but sharply expanding posteriorly. The frontoparietal expands dorsolaterally to form a low frontoparietal crest, which is heavily exostosed, especially anteriorly.

#### Ventral investing and palatal bones (Figs. 7, 8)

The parasphenoid is nib-shaped, forming the floor of the braincase. The pointed cultriform process overlaps the sphenethmoid ventrally. The parasphenoid alary processes provide the floor for the otic capsules and are approximately perpendicular to the cultriform process. The posteromedial process of the parasphenoid is broadly acuminate and reaches the margin of the foramen magnum. The lateral arms of the parasphenoid are broadly in contact with the long medial ramus of the pterygoid. The massive neopalatine is fused posteromedially to the sphenethmoid, the vomers are wide and lack postchoanal processes. Postchoanal vomers are straight, clearly distinguishable, anterior to the neopalatine, which they do not contact. Each vomer bears ca. 9 teeth. The neopalatine is not fused to the inner surface maxilla.

#### Maxillary arcade (Figs. 7, 8, 9)

Both maxillae and premaxillae are dentate, and mostly exostosed. The premaxillae are separated medially, inclined posteriorly. The alary processes of the premaxillae are broad and acuminate posteriorly, diverging from the midline. The alary processes are directed posterodorsally and have a deep indentation along their anteromedial base. In dorsal view the alary processes reach the level of the anteriormost margin of the maxilla. The palatine (medial) process of the premaxilla is short, pointed, directed posterodorsally. The lateral process of the premaxilla is short, similar in size to the medial process, pointed and directed posterolaterally. The premaxillae are not fused to the maxillae. The maxilla is greatly expanded, the pars facialis is well developed but not fused with the maxillary process of the nasal. Anteriorly and in lateral view, the maxilla is squarish and almost twice as high as it is posteriorly. The maxilla possesses a short posterodorsal projection directed towards the zygomatic ramus of the squamosal with which it is in close contact, but not fused. Posteriorly, the maxilla is contacting, but not fused to the robust quadratojugal.

#### Suspensory apparatus (Fig. 7)

The triradiate pterygoid is robust. The anterior ramus extends toward the braincase from the maxilla at the mid-orbit level and braces against the anteroventral margin of the otic capsule via the long medial ramus. The posterior ramus is broad and flat and appears to be in tight contact with the ventral ramus of the squamosal. The posterior and medial rami are of approximately equal length. The quadratojugal is robust, in contact with but not fused to the maxilla. The otic and ventral rami of the squamosal are well developed; the otic ramus extends over the lateral margin of the prootic and onto its dorsal surface. The ventral ramus of the squamosal is narrow in lateral view and extends from the quadratojugal to the posterodorsal margin of the orbit. The otic ramus bears a low crest laterally, is heavily exostosed and is approximately half the length of the narrower zygomatic ramus. The zygomatic ramus is long and acuminate in lateral profile, in contact with but not fused to the postorbital process of the maxilla. The zygomatic rami do not appear exostosed.

#### Mandible (Fig. 7)

The dentary is long and stout, posteriorly acuminate, fused to the small, arcuate mentomeckelian bone anteriorly. The mentomeckelians are separated medially. The dentary overlaps the angulosplenial for nearly one third of the angulosplenial length. The main component of the mandible is the angulosplenial, which is long and weakly sigmoid, acuminate anteriorly, and extends nearly to the mentomeckelian anteriorly. The coronoid process is dorsomedial and well-developed, about 1/4 of the posterior ramus. The only part of the hyoid revealed are the posteromedial processes, which are fused anterodistally and slightly expanded proximally. There is no mineralization in the hyoid corpus.

### Postcranium (Fig. 6)

The vertebral column is composed of 8 nonimbricate, procoelous presacral vertebrae, sacrum, and urostyle. The atlantal cotylar arrangement corresponds to the Type I of Lynch [32]. Presacrals I–III expand dorsally. The neural arches are well developed and bear small projecting neural crests on presacrals II–III. The transverse processes are moderately elongated, distally expanded on presacral II–IV. The length of the transverse processes is III > IV > V = VI = VII = VIII > II. The transverse processes of presacral II are directed roughly perpendicularly and ventrally to the medial axis; those of presacral III are directed posteroventrally; transverse processes of presacral IV to VII are directed posterodorsally; and those of presacral VIII are directed perpendicular and dorsally to the medial axis. Presence of paired calcified processes (likely calcified endolymphatic sacs [33]) extending through the intervertebral foramina, but not investing the ventral face of the transverse processes of the vertebrae. These processes project posteriorly along the ilium as two protuberances almost reaching the posterior margin of the tuber superius in lateral view. The sacral diapophyses are slightly flattened, slightly expanded distally and of similar length as the transverse processes of presacral III. The sacral diapophyses are directed posterodorsally, with truncate distal borders and are not in contact with the ilia distally. The sacrum has a bicondylar articulation with the urostyle. The urostyle is about the same length as the presacral vertebral column, with its posterior tip upturned. It bears a well-developed dorsal crest along just over half of its shaft. The crest starts anteriorly as a large, ossified tubercle and progressively decreases in height in the caudal direction. The pectoral girdle is arciferal. The clavicles are robust, flattened, arcuate, directed anteriorly, and moderately separated from one another medially; the clavicle appears to be fused to the scapula and attached to the coracoid. The posterior margin of the stout coracoid is weakly sigmoid, whereas the anterior margin is concave. The coracoids are separated and expanded medially, the concave glenoid and convex sternal ends are about equally expanded, almost three times as wide as the midshaft width of the bone. The pars acromialis of the scapula appears to be paired with the pars glenoidalis. The cleithrum is a dagger-shaped element, the suprascapular cartilage appears to not be ossified. The head of the humerus is ossified. There is a moderate crista ventralis extending along the proximal half of the bone. The cristae medialis and lateralis are visible in ventral (flexor) view. The capitulum and ulnar and radial condyles appear to be well developed. The olecranon of the radio-ulna is round, the sulcus intermedius is indicated by a distinct groove; the epiphyses of the radius and ulna appear to be ossified, as well as all carpal elements and the prepollex. The carpus is composed of a radiale, ulnare, ossified prepollex element, element Y, distal carpal 2 and an element representing the fusion of distal carpals 3–5 (dcpl 3–5). Both ulnare and dcpl 3–5 display a lateral apophysis on their dorsal border. The finger phalangeal formula is standard (2–2-3–3), and the metacarpals increase in size in the following order: II, I, IV, and III. The relative lengths of the fingers increase in size in the following order: II, I, IV, and III. Presence of a distal process on the third metacarpal (Fig. 6). The phalangeal elements are well ossified, the distal phalanges are slightly curved downwards with a pointed tip. The postsacral trunk region is relatively short and narrow. The articulation between the anterior end of the ilial shafts and the ventral side of the distal ends of the sacral transverse processes is of the sagittal-hinge type [34], usually characteristic of long-distance jumpers. The ilial shafts have large crests along almost their full length, originating approximately at the level of the urostyle tubercle and terminating in a posterior prominence. The ilia are posteriorly fused to the ischium. The pubis is almost completely ossified; the acetabulum is round, well developed. The femur is distinctly shorter than the tibiofibula. The femur is weakly sigmoid and bears a posteroventral ridge on its proximal end. The sulcus intermedius of the tibiofibula is much less prominent than the sulcus intermedius of the radio-ulna. The astragalus and calcaneum are about two-thirds the size of the tibiofibula. These structures are widely separated at their midpoint and fused at their distal and proximal heads. Two tarsals (distal tarsal 1 and an element representing the fusion of distal tarsals 2–3) are present at the base of Toes II and III. A small element Y and a short ossified prehallux element are also present, at the base of Toe I. The toe phalangeal formula is standard (2–2-3–4-3), and the metatarsals increase in size in the following order: I, II, III, V, IV. The relative lengths of the toes increase in the same order. The phalangeal elements are well ossified, the ultimate phalange of the toes appears to be similar in shape and size to that of the fingers.

### Variation

**Fig. 10:**
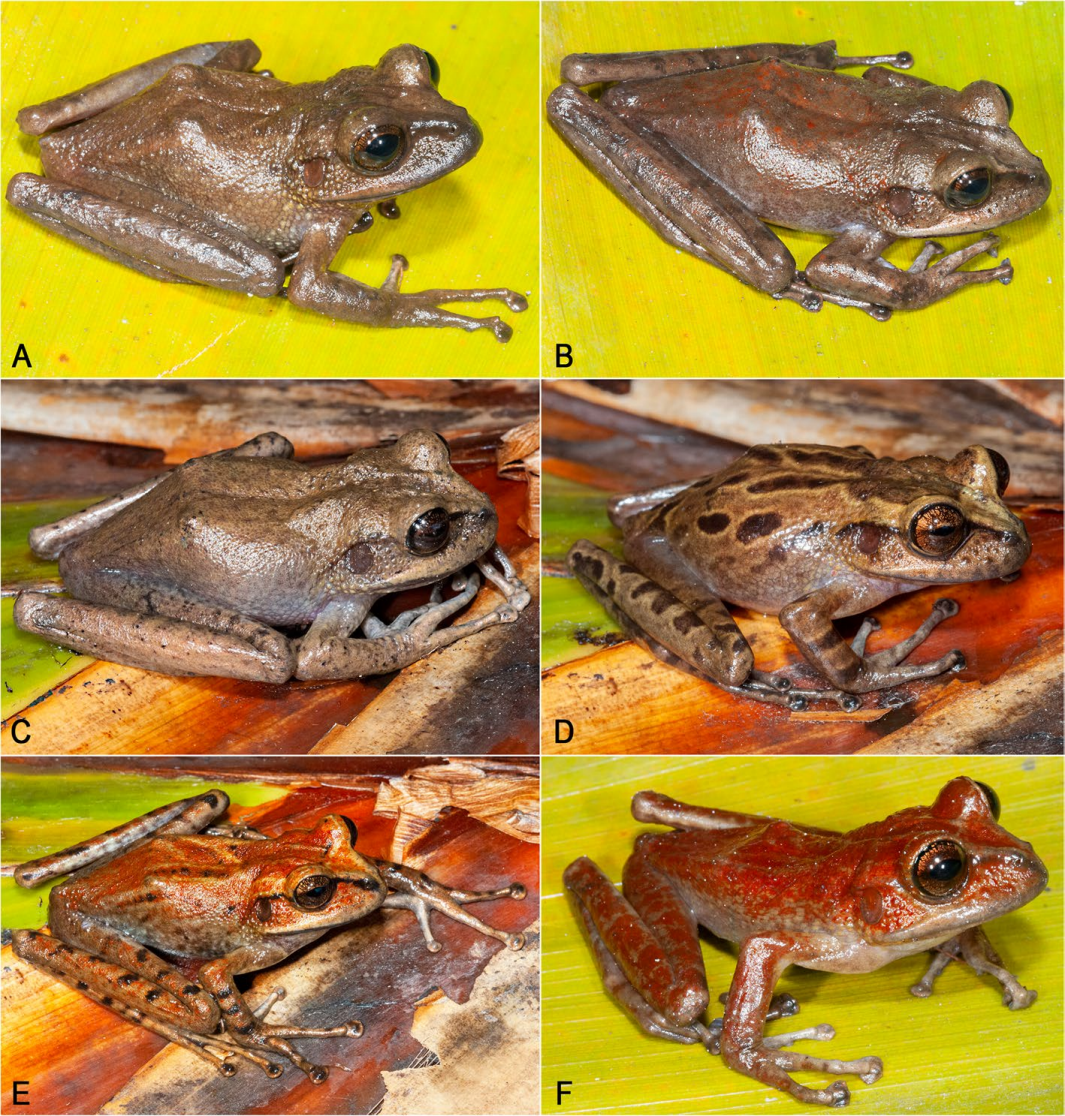
Main color pattern variation in *Stefania maccullochi* **sp. nov. A** IRSNB 15854, male. **B** NHMUK 2023.3188 (PK2098), female. **C** IRSNB 15855, female. **D** NHMUK 2023.3193 (PK2148), male. **E** NHMUK 2023.3192 (PK2137), male. **F** NHMUK 2023.3187 (PK2084), male. Photos by the author

*Stefania maccullochi* **sp. nov.** is a highly polychromatic species (Fig. 10). Polychromatism was not found to be related to sex. Adult individuals range from plain medium brown, plain light grey-brown, medium brown with some more or less extensive red wash on the dorsal surfaces, chestnut brown with dark brown markings outlined by cream color (a pattern reminiscent of *S. ayangannae*, although less contrasted and without conspicuous dark brown bars on flanks and lips), to almost completely red. Dorsal markings greatly vary as well, from none to a few faint chevrons and conspicuous spots. In life, a narrow (broad in one specimen of the 23 examined) canthal dark brown stripe is present in all specimens (although not visible in preservative); an interorbital bar is sometimes present. The upper surface of limbs may be plain or with faint to conspicuous dark brown spots and/or bars. The dorsal skin is shagreened, with or without a few sparse enlarged tubercles. Males are distinctly smaller than females: 45.7–54.6 mm SVL in adult males (*n* = 12) vs 55.4–72.9 mm SVL in adult females (*n* = 9). Head is slightly longer than wide, or slightly wider than long. The number of vomerine teeth varies between 3–9. There is no significant difference in toe webbing between the sexes; toe webbing formula for all adult males (*n* = 12) is I 3—3 II (2^+^–2)—(3 ½–3^+^) III (2 ¾–2^+^)—(4^+^–4) IV (3 ¾–3 ½)—(2 ½–2^+^) V; toe webbing formula for all adult females (*n* = 9) is I 3—3 II (2^+^–2)—(3 ¾–3 ¼) III (2 ½–2^+^)—(4–3 ¾) IV (3 ¾–3 ½)—(2^+^–2) V. None of the 12 males examined has detectable nuptial excrescences.

### Molecular divergences within the *Stefania riveroi* clade

Based on the same fragment of 16S ribosomal RNA gene used in Kok et al. [6], genetic distances between *S. riveroi* and *S. maccullochi* **sp. nov.** are 4.3–4.7% (allopatric species). Genetic distances between *S. ayangannae* and *S. maccullochi* **sp. nov.** are 4.1–4.7% (allopatric species). Genetic distances between *S. coxi* and *S. maccullochi* **sp. nov.** are 4.5–4.9% (allopatric species). By comparison, genetic distances between *S. ayangannae* and *S. riveroi* are 2.9–3.1% (allopatric species), genetic distances between *S. coxi* and *S. ayangannae* are 4.7–4.9% (sympatric/syntopic species). Intraspecific divergences within the clade vary from 0% (including between allopatric populations of *S. coxi* from Mount Ayanganna and Mount Wokomung) to 0.2% between allopatric populations of *S. ayangannae* from Mount Ayanganna and Mount Wokomung (Table 1, Fig. 2).

**Table 1:** Genetic distances in the barcoding fragment of 16S rRNA (511 base pairs) within the *Stefania riveroi* clade

		**1**	**2**	**3**	**4**	**5**	**6**	**7**	**8**	**9**	**10**
1	*Stefania coxi* (ROM 42856) Mount Wokomung, Guyana										
2	*Stefania coxi* (ROM 39478) Mount Ayanganna, Guyana	0.000									
3	*Stefania ayangannae* (ROM 39640) Mount Ayanganna, Guyana	0.049	0.049								
4	*Stefania ayangannae* (ROM 42925) Mount Wokomung, Guyana	0.047	0.047	0.002							
5	*Stefania ayangannae* (ROM 42906) Mount Wokomung, Guyana	0.047	0.047	0.002	0.000						
6	*Stefania maccullochi* **sp. nov.** (IRSNB 15853) Wei-Assipu-tepui, Guyana	0.045	0.045	0.043	0.041	0.041					
7	*Stefania maccullochi* **sp. nov.** (IRSNB 15855) Wei-Assipu-tepui, Guyana	0.049	0.049	0.047	0.045	0.045	0.000				
8	*Stefania maccullochi* **sp. nov.** (IRSNB 15854) Wei-Assipu-tepui, Guyana	0.045	0.045	0.043	0.041	0.041	0.000	0.000			
9	*Stefania riveroi* (IRSNB 15703) Yuruaní-tepui, Venezuela	0.049	0.049	0.031	0.029	0.029	0.043	0.047	0.043		
10	*Stefania riveroi* (IRSNB 15716) Yuruaní-tepui, Venezuela	0.049	0.049	0.031	0.029	0.029	0.043	0.047	0.043	0.000	

### Distribution and natural history

*Stefania maccullochi* **sp. nov.** is seemingly endemic to the summit of Wei-Assipu-tepui at the border between Guyana and Brazil (Fig. 2). The genus *Stefania* is absent from the higher summits of Roraima-tepui (ca. 2800 m elevation) and Kukenán-tepui (ca. 2600 m elevation) [35, 36] (pers. obs.), and I did not find it on the summit of Maringma-tepui (ca. 2100 m elevation), which I explored for 5 days in November 2007. Neither did D. B. Means (pers. comm.) during 6 days on Maringma-tepui in February 2006. *Stefania maccullochi* **sp. nov.** was never found at lower elevations in the intervening uplands in the Pakaraima Mountains, where other *Stefania* species occur [6] (pers. obs.). D. B. Means (pers. comm.) did not find the species during any of his five expeditions in the cloud forest between Roraima-tepui and Wei-Assipu-tepui, nor on the lower slopes of Wei-Assipu-tepui.

**Fig. 11:**
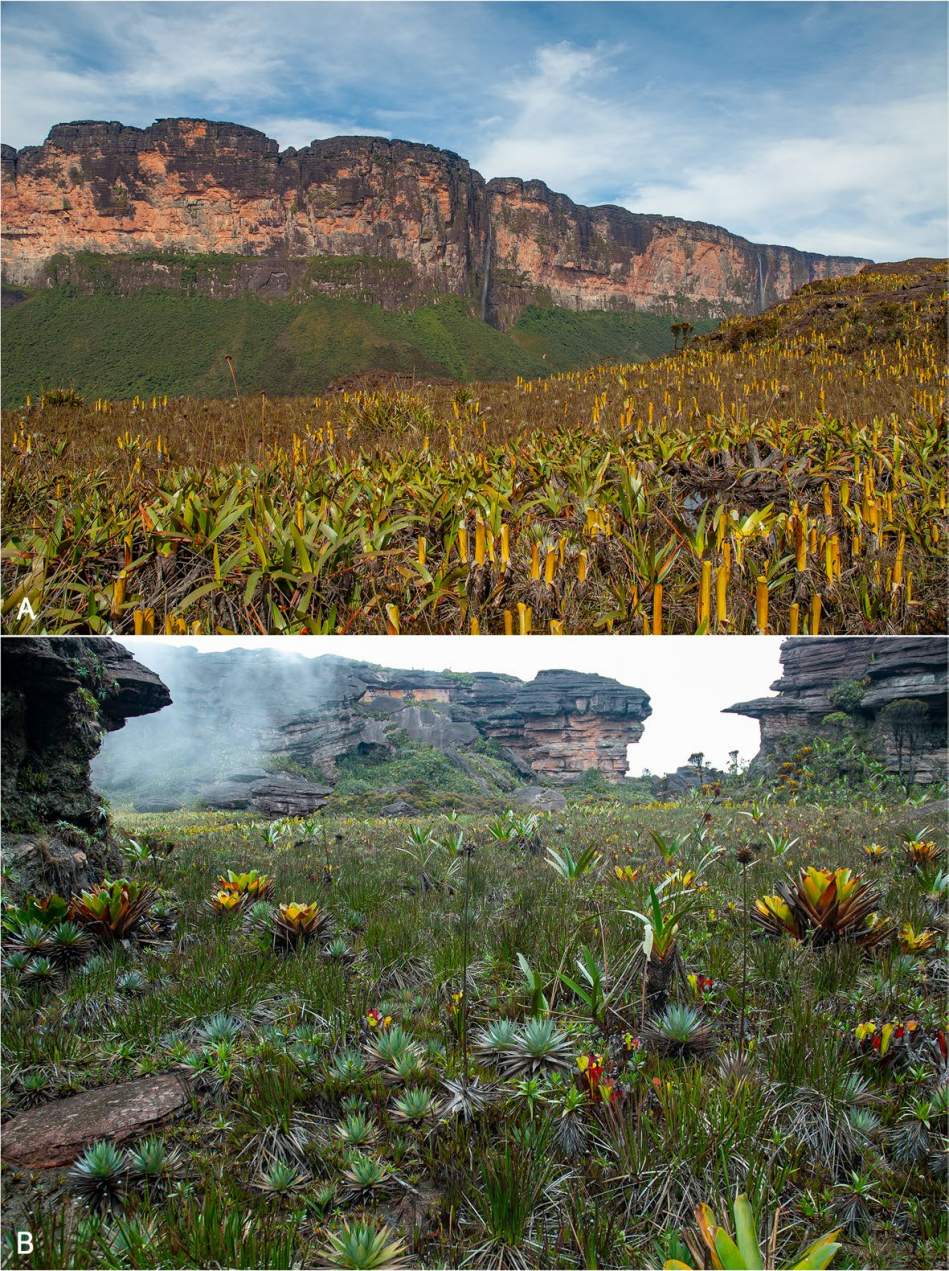
Main macrohabitats on the summit of Wei-Assipu-tepui. The cliff visible in the background of **A** is the eastern flank of Roraima-tepui. Photos by the author

Wei-Assipu-tepui is richly vegetated on most of its summit, which is covered by extensive areas of coarse herbs mixed with woody subshrubs on peat soils [4]. Some quaking bogs and extensive patches of dwarf forests dominated by *Bonnetia roraimae* (Theaceae) also occur. The terrestrial bromeliads *Brocchinia tatei* and *B. reducta* (Bromeliaceae) as well as *Stegolepis guianensis* (Rapateaceae), *Orectanthe sceptrum* (Xyridaceae), and *Heliamphora nutans* (Sarraceniaceae) are especially abundant [4] (Fig. 11). A maximum temperature of 29 °C (during the day, on exposed rock), a minimum temperature of 11 °C (at night, under large boulders), and a relative hygrometry varying from 24% (day) to 98% (night) were recorded in November 2009 [4].

**Fig. 12:**
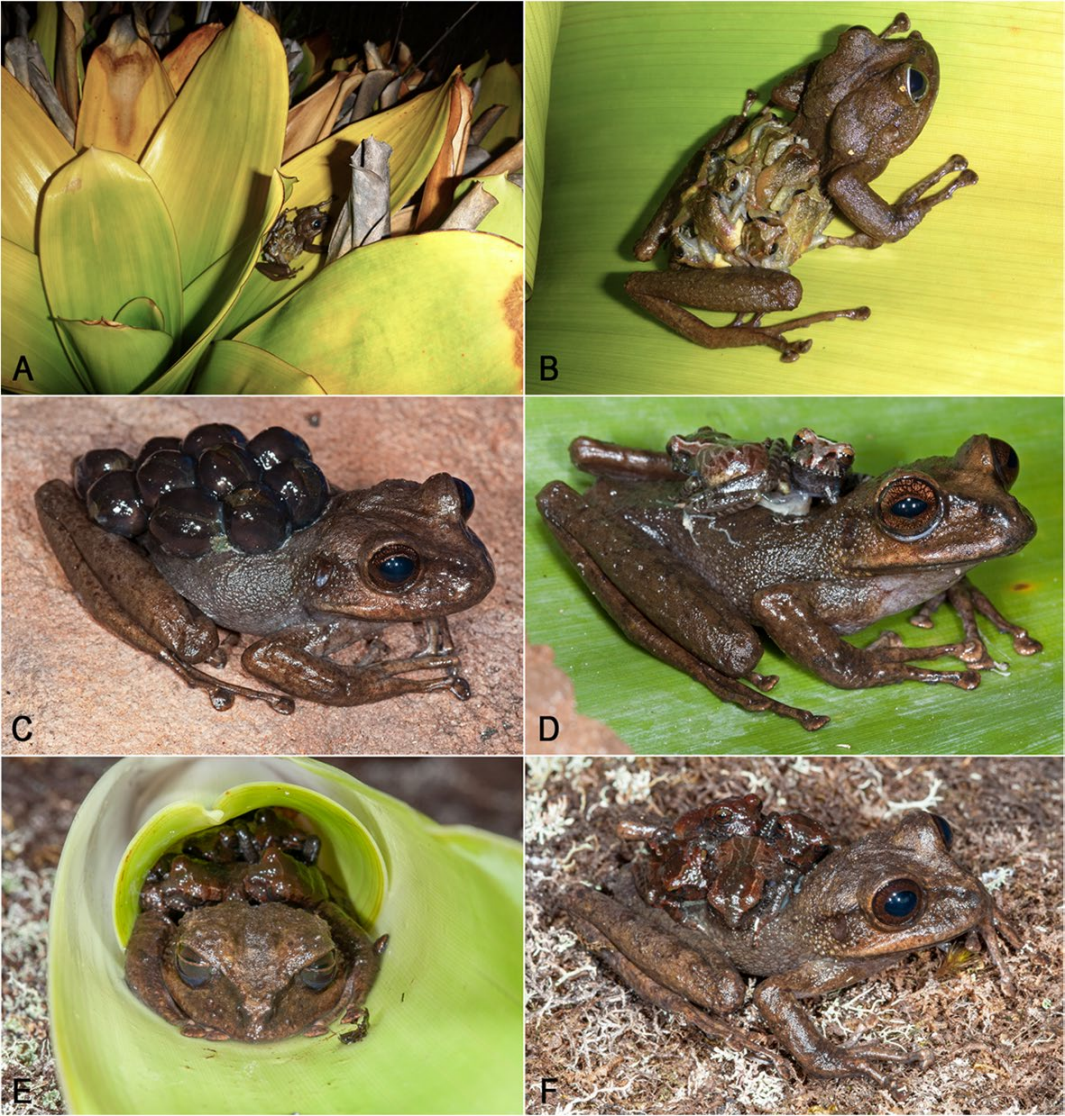
Various females of *Stefania maccullochi* **sp. nov.** carrying eggs and juveniles. **A **and** B**. Uncollected female carrying 9 juveniles. **C**. NHMUK 2023.3190 (PK2122), carrying 10 eggs/metamorphs. **D**. NHMUK 2023.3185 (PK2063), carrying 2 near-term juveniles. **E and F**. IRSNB 15853, carrying 4 near-term juveniles. Photos by the author

Active specimens of *Stefania maccullochi* **sp. nov.** were exclusively found at night, usually moving on the ground or in low vegetation (including a female carrying 10 eggs). An uncollected female carrying nine juveniles was observed at night in a *Brocchinia micrantha* (Fig. 12). Two females carrying near-term juveniles (two and four, respectively) were found during the day, hiding inside the tube of *Brocchinia reducta* (head up, see Fig. 12). Another female carrying nine juveniles was collected resting under a rock during the day. Other specimens observed during the day were found resting in cracks between rocks (five individuals), on small trees between 50–200 cm above the ground (four individuals), and on the ground among vegetation (five individuals). One individual was found sitting on a mossy rock at the bottom of a 30 m-deep crevice. No male was observed calling, although a loud single note call attributed to a *Stefania* was sometimes heard from inside shallow crevices (not recorded).

### Amended definitions for the three other known species in the *Stefania riveroi* clade

**Fig. 13:**
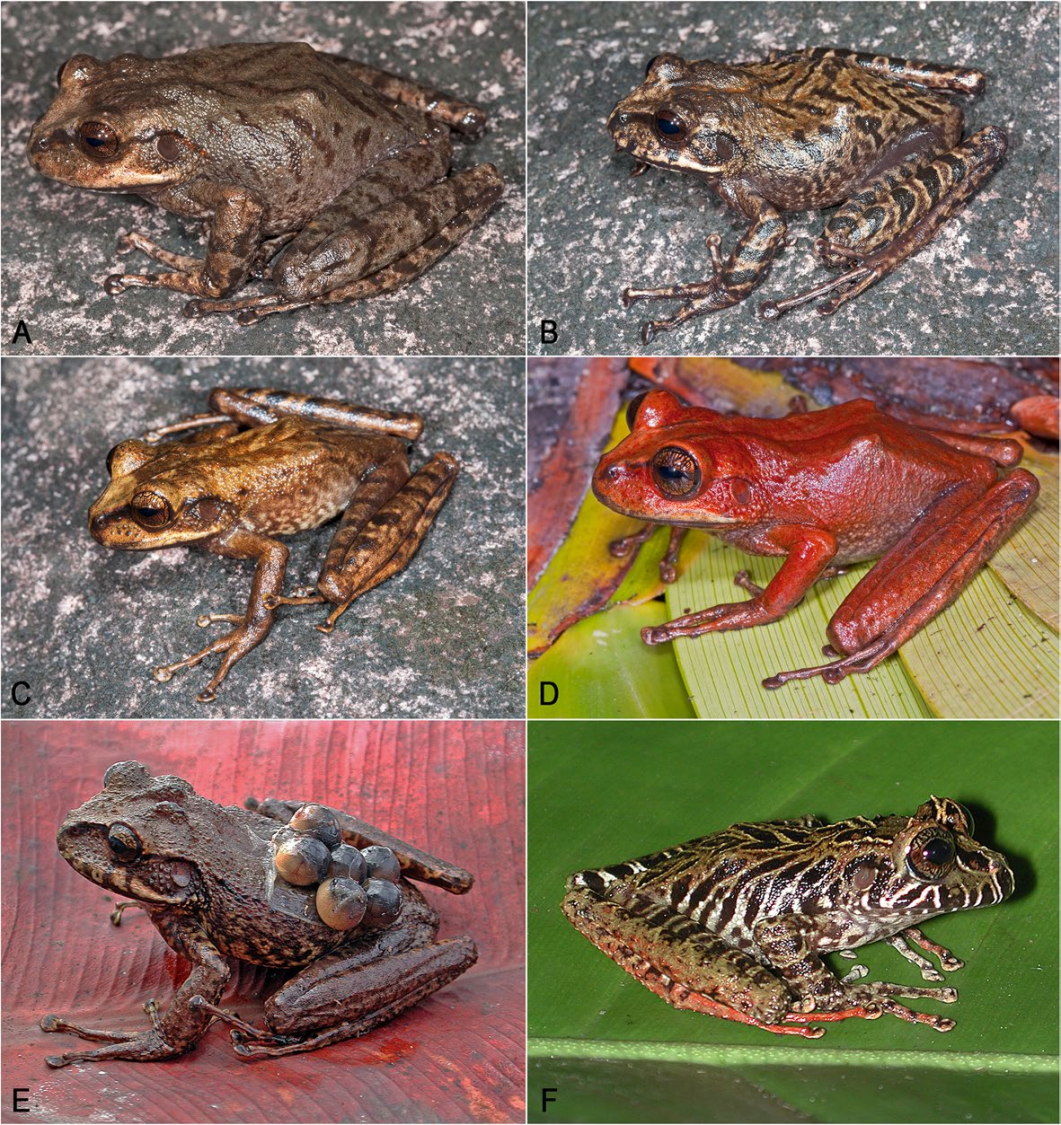
Main color pattern variation in *Stefania riveroi*, and illustration of the other known species in the *Stefania riveroi* clade*.* **A**. *S. riveroi*, IRSNB 15719, female. **B**. *S. riveroi*, IRSNB 15715, male. **C**. *S. riveroi*, IRSNB 15703, male. **D**. *S. riveroi*, IRSNB 15740, female. **E**. *S. coxi*, uncatalogued, female carrying 6 eggs. **F**. *S. ayangannae*, uncatalogued/unsexed. Photos by the author, except **E** and **F** by D. Bruce Means

***Stefania ayangannae*** (Fig. 13) is characterized by the combination of the following morphological characters: (1) a small species of *Stefania*, max SVL in preserved females 54.8 mm, 46.5 mm in preserved males; (2) head not distinctly longer than wide, about as wide as long; (3) canthus rostralis slightly granular/tuberculate, prominent, angular, concave, canthal stripe present in life, sometimes inconspicuous; (4) loreal region with a few low tubercles; (5) upper eyelid with scattered tubercles, presence of an enlarged triangular appendage on its posterior upper part; (6) frontoparietal ridges conspicuous, medium in height (in life/preservative); (7) frontoparietal crests present, moderately developed, projecting dorsally (on cranium); (8) absence of distinct constriction of the frontoparietal bones; (9) absence of extensive exostosis on the cranium (seemingly limited to nasals, lateral edges of frontoparietals, and zygomatic and otic rami of the squamosal); (10) premaxillae inclined posteriorly; (11) posterodorsal projection of maxilla in contact with orbital/zygomatic ramus of squamosal; (12) maxillary process of the nasal in contact with the maxilla; (13) horizontal length of tympanum less than 50% horizontal length of eye in both sexes; (14) vomerine teeth 2–7; (15) toes II–V basally webbed, no significant difference in toe webbing between sexes; (16) dorsal skin (in life) shagreened, with or without a few sparse enlarged tubercles; (17) ventral skin (in life) areolate; (18) presence of conspicuous outer tarsal tubercles (in life); (19) presence of multiple conspicuous dark brown bars on flanks and lips, absence of dorsolateral stripes (in life); (20) in living adults, iris unicolor (a dark brown horizontal band is sometimes present), golden brown to copper, with extensive dark brown reticulations.

***Stefania coxi*** (Fig. 13) is characterized by the combination of the following morphological characters: (1) a large species of *Stefania*, max SVL in preserved females 70.0 mm, 51.0 mm in preserved males; (2) head wider than long; (3) canthus rostralis slightly granular/tuberculate, prominent, angular, straight or weakly sigmoid, canthal stripe present in life; (4) loreal region with numerous low tubercles; (5) upper eyelid with scattered tubercles, absence of an enlarged triangular appendage on its posterior upper part; (6) frontoparietal ridges conspicuous, hyper developed (in life/preservative); (7) frontoparietal crests present, hyper developed, projecting dorsally (on cranium); (8) constriction of the frontoparietal bones anterior to the anterior epiotic eminence; (9) extensive exostosis on the cranium; (10) premaxillae inclined posteriorly; (11) posterodorsal projection of maxilla in contact with orbital/zygomatic ramus of squamosal; (12) maxillary process of the nasal in contact with the maxilla; (13) horizontal length of tympanum more than 50% horizontal length of eye in both sexes; (14) vomerine teeth 3–6; (15) toes one-third webbed, no significant difference in toe webbing between sexes; (16) dorsal skin (in life) smooth to finely shagreened, with scattered tubercles; (17) ventral skin (in life) finely granular with scattered tubercles; (18) presence of conspicuous outer tarsal tubercles (in life); (19) presence of conspicuous dark brown bars on lips, but not on flanks, absence of dorsolateral stripes (in life); (20) in living adults, iris bicolor, tan above, reddish brown below, with extensive dark brown reticulations.

***Stefania riveroi*** (Fig. 13) is characterized by the combination of the following morphological characters: (1) a large species of *Stefania*, max SVL in preserved females 70.0 mm, 59.0 mm in preserved males; (2) head not distinctly longer than wide, about as wide as long; (3) canthus rostralis smooth, prominent, rounded, concave, canthal stripe present in life except in plain red individuals; (4) loreal region with a few low tubercles; (5) upper eyelid mostly smooth, absence of enlarged triangular appendage on its posterior upper part, although a conical tubercle may be present medially; (6) frontoparietal ridges conspicuous, low (in life/preservative); (7) frontoparietal crests present, feebly developed, laterally projecting (on cranium); (8) constriction of the frontoparietal bones at the level of the anterior epiotic eminence; (9) absence of extensive exostosis on the cranium; (10) premaxillae inclined posteriorly; (11) posterodorsal projection of maxilla fused with orbital/zygomatic ramus of squamosal; (12) maxillary process of the nasal fused with the maxilla; (13) horizontal length of tympanum more than 50% horizontal length of eye in both sexes; (14) vomerine teeth 2–8; (15) Toes II–V basally webbed, no significant difference in toe webbing between sexes; (16) dorsal skin (in life) shagreened to finely granular, with or without a few sparse enlarged tubercles; (17) ventral skin (in life) granular; (18) absence of conspicuous outer tarsal tubercles (in life); (19) absence of multiple conspicuous dark brown bars on flanks and lips, absence of dorsolateral stripes (in life); (20) in living adults, iris unicolor, copper, with extensive dark brown reticulations.

## Discussion

Multilocus genetic analyses of the genus *Stefania* have indicated that species boundaries and phylogenetic relationships are often incongruent with morphological traits in this clade [6, 37]. *Stefania riveroi* and *S. maccullochi* **sp. nov.** are not reciprocally monophyletic but are morphologically extremely similar. Both species are polychromatic (Figs. 10, 13) making them exceedingly hard to differentiate based on phenotype alone. The morphological similarity between *Stefania riveroi* and *S. maccullochi* **sp. nov.** could be a consequence of shared ancestry (symplesiomorphy/morphological stasis) or a result of convergent or of parallel evolution (homoplasy). The fact that these two isolated species inhabit similar tepui summit habitats under seemingly similar ecological pressure and constraints seems in favor of homoplasy (both tepui summit surfaces are less than 5 km^2^ and less than 2500 m elevation), but further research is needed to disentangle these two possible explanations. A probable scenario is a combination of both hypotheses having acted synergistically. Given the divergence times within the *S. riveroi* clade (no split between species younger than the Miocene [6]) and current isolation of *S. maccullochi* **sp. nov.**, the hypothesis that hybridization could be responsible for the discrepancy between morphology and molecular data is less credible. A similar scenario occurs in the *S. ginesi* clade found in the Chimantá Massif and peripheral tepuis [6, 25, 37], which was found to contain seven isolated tepui summit species (most of them cryptic) instead of two species widespread on multiple tepui summits.

Phylogenetic position, genetic divergences and geographic distribution leave no doubt that *S. maccullochi* **sp. nov.** is a separately evolving metapopulation lineage (i.e., species according to the unified species concept [38]). Genetic divergences in 16S rRNA between *S. riveroi* and *S. maccullochi* **sp. nov.** (4.3–4.7%) strongly exceed intraspecific genetic divergence in all species of the *S. riveroi* clade, even when comparing allopatric populations (0–0.2%) (see above and Table 1).

Interpretation of µCT-scan reconstructions may be biased by factors such as decalcification of the specimen examined, resolution (voxel size), possible operator errors during segmentation, and segmentation artifacts, especially when working on extracted volume meshes. The subtle osteological characters distinguishing *S. maccullochi* **sp. nov.** from *S. riveroi* are not immune to these issues and should be confirmed by the analysis of a larger number of specimens scanned at similar resolution. Distinctions between contact and fusion of specific elements reported in this study could merely reflect different resolutions of the scans (see Additional file 1: Table S1 for details). If these potential characters are not constant enough to be considered diagnostic, then there might be virtually no morphological character to separate *S. maccullochi* **sp. nov.** from *S. riveroi*.

Skin texture and markings are often affected by preservation (especially long-term) and may be challenging to properly assess on preserved specimens alone. Likewise, the shape of some ridges, such as the canthus rostralis may be altered (smoothened) by a long stay in ethanol preservative. Hence the importance of taking photographs and notes about specimens in life. In *Stefania*, as in other frogs, outer metatarsal and loreal tubercles are prone to disappear in preservative, and the condition of the dorsal and ventral skin may also be altered by preservation, leading to inaccurate descriptions.

To the best of my knowledge, the presence of a distal process on the third metacarpal had never been reported in *Stefania* [7, 19–21, 27, 39, 40]. I found this process (Fig. 6)—comparable with the medial process on the third metacarpal in Centrolenidae [41]—in all species examined (except in individuals with decalcified digits), including outside the *S. riveroi* clade, and this condition is suggested as a new synapomorphy of the genus. The distal process on the third metacarpal appears to be more developed in some species than in others, but a larger sampling is necessary to confirm this and exclude µCT scan segmentation artifacts as the origin of size differences.

In accordance with criteria B1 and B2a,b [42], i.e., an extent of occurrence < 100 km^2^, an area of occupancy < 10 km^2^, a single known location, and projected decline—due to increasing anthropogenic fires in the region and effects of climate change [43]—*S. maccullochi* **sp. nov.** should be listed as Critically Endangered. Owing to its current strong isolation the species is less prone to human-mediated introduction of pathogens, such as chytridiomycosis, but recent incursions for documentary filming [44] might have put the species at risk if adequate disinfection protocols have not been followed [45].

## Conclusions

*Stefania maccullochi* **sp. nov.** is a microendemic and morphologically/taxonomically cryptic species belonging to the *S. riveroi* clade. The *S. riveroi* clade, as presently understood, is restricted to the highlands and uplands (1200–2400 m elevation) of the Eastern Tepui Chain in Venezuela/Guyana/Brazil, and the Pakaraima Mountains in western Guyana. The new species is only known from the small summit (ca. 3 km^2^) of Wei-Assipu-tepui, an isolated table-top mountain at the border between Guyana and Brazil. *Stefania maccullochi* **sp. nov.** is considered critically endangered according to IUCN criteria. Although morphologically highly similar to *S. riveroi* from the summit of Yuruaní-tepui, a table-top mountain in Venezuela, *S. maccullochi* **sp. nov.** has been shown to be sister to *S. ayangannae*, a species occurring on two major tepuian massifs in western Guyana. *Stefania maccullochi* **sp. nov.** and *S. ayangannae* are easily diagnosed by size, osteology, skin texture, and color pattern. The striking morphological similarity between *S. riveroi* (sister to the three other species in the *S. riveroi* clade) and *S. maccullochi* **sp. nov.** is probably a consequence of symplesiomorphy and/or homoplasy. Examination of the skeletal morphology of the new species and comparison with congeners highlighted a previously unnoticed osteological synapomorphy for the genus: the presence of a distal process on the third metacarpal. This description brings the number of described *Stefania* species to 20, all (near-) endemic taxa inhabiting the biodiverse Pantepui biogeographical region.

## Supplementary Information

**Additional file 1: Table S1.** µCT scan data for the holotype of *Stefania maccullochi* **sp. nov.** and for comparative species in the *S. riveroi* clade.

**Additional file 2: Appendix.** List of additional museum specimens examined.

## Data Availability

Further information and requests for additional resources should be directed to and will be fulfilled by the author.

## Abbreviations

Ang: Angulosplenial
ang.cp: Coronoid process of the angulosplenial
col: Columella
c.p: Cultriform process
d: Dentary
dcpl: Distal carpal
exo: Exoccipital
FIII: Finger III
FIV: Finger IV
Fp: Frontoparietal
lam.p: Lamina perpendicularis
mmk: Mentomeckelian
mx: Maxilla
mx.pp: Posterodorsal projection of the maxilla
na: Nasal
na.mp: Maxillary process of the nasal
neo: Neopalatine
p.f: Pars facialis
pmx: Premaxilla
pmx.ap: Alary process of the premaxilla
pmx.lp: Lateral process of the premaxilla
pmx.pp: Palatine process of the premaxilla
pro: Prootic
psp: Parasphenoid
psp.ar: Alary process of the parasphenoid
psp.pp: Posteromedial process of the parasphenoid
pt: Pterygoid
pt.ar: Anterior ramus of the pterygoid
pt.mr: Medial ramus of the pterygoid
pt.pr: Posterior ramus of the pterygoid
qua: Quadratojugal
rRNA: Ribosomal RNA
smx: Septomaxilla
sph: Sphenethmoid
squa: Squamosal
squa.or: Otic ramus of the squamosal
squa.vr: Ventral ramus of the squamosal
squa.zr: Zygomatic ramus of the squamosal
SVL: Snout-vent length
TI: Toe I
TII: Toe II
V: Vomer
