# Supplementary material for: Bones and all: a new critically endangered Pantepui species of Stefania (Anura: Hemiphractidae) and a new osteological synapomorphy for the genus
Source: Zoological Lett. 2023 May 25;9:11. doi: 10.1186/s40851-023-00209-6 (PMC10210337; doi:10.1186/s40851-023-00209-6)
Supplement: Supplementary file 1 — Additional file 1: Table S1. µCT scan data for the holotype of Stefania maccullochi sp. nov. and for comparative species in the S. riveroi clade. [file 40851_2023_209_MOESM1_ESM.docx]

**Table S1.** µCT scan data for the holotype of *Stefania maccullochi* **sp. nov.** and for comparative species in the *S. riveroi* clade.

| **Species** | **Museum n°** | **Field n°** | **Sex** | **Resolution (voxel size)** | **Source** |
| --- | --- | --- | --- | --- | --- |
| *Stefania maccullochi* **sp. nov.** | NHMUK 2023.3184 | PK2071 | F | 27.7 µm | <https://www.morphosource.org/concern/media/000515082> |
| *Stefania ayangannae* | CPI 10656 | NA | F | 38.0 µm | <https://sketchfab.com/3d-models/stefania-sp-10656-54fcac29612c4e7db5c45b85b48f4437> |
| *Stefania ayangannae* | CPI 10657 | NA | F | 43.0 µm | <https://sketchfab.com/3d-models/stefania-sp-cpi10657-047d0bd6070d46728644d5100a813730> |
| *Stefania coxi* | ROM 39479 | ROM35155 | M | 44.0 µm | <https://sketchfab.com/3d-models/stefania-sp-39479-35155-2b3198b2582c43b2b4221f5d1e0c4382> |
| *Stefania coxi* | ROM 39480 | ROM35025 | F | 55.5 µm | <https://sketchfab.com/3d-models/stefania-sp-39480-35025-ef367f89b7654e6187587fd119da61ac> |
| *Stefania riveroi* | IRSNB 15727 | PK2294 | M | 49.5 µm | <https://sketchfab.com/3d-models/stefania-sp-pk2294-225644d27fb5406f846a2a9e554268da> |
| *Stefania riveroi* | IRSNB 15730 | PK2297 | F | 50.0 µm | <https://sketchfab.com/3d-models/stefania-sp-pk2297-9e9b67de0dd749a7a98cba15d5a5b8cb> |
