# Supplementary material for: Bones and all: a new critically endangered Pantepui species of Stefania (Anura: Hemiphractidae) and a new osteological synapomorphy for the genus
Source: Zoological Lett. 2023 May 25;9:11. doi: 10.1186/s40851-023-00209-6 (PMC10210337; doi:10.1186/s40851-023-00209-6)
Supplement: Supplementary file 2 — Additional file 2: Appendix. List of additional museum specimens examined. [file 40851_2023_209_MOESM2_ESM.docx]

**APPENDIX – List of additional museum specimens examined.** Institution acronyms are as follows: AMNH = American Museum of Natural History, New York, USA; CPI = Coastal Plains Institute and Land Conservancy, Tallahassee, USA; IRSNB = Royal Belgian Institute of Natural Sciences, Brussels, Belgium; MBUCV = Universidad Central de Venezuela, Caracas, Venezuela; MHNLS = Museo de Historia Natural La Salle, Caracas, Venezuela; ROM = Royal Ontario Museum, Toronto, Canada.

***Stefania riveroi* clade**

*Stefania ayangannae* (n = 45): CPI 10473–10474, CPI 10656–10657 (including µCT scans), CPI 10818–10830, CPI 10911, CPI 10986–10992, CPI 11030–11032, CPI 11040–11041, CPI 11070–11085, Mount Wokomung, Cuyuni-Mazaruni, Guyana.

*Stefania coxi* (n = 3): ROM 39479–39480 (paratypes, including µCT scans), Mount Ayanganna, Cuyuni-Mazaruni, Guyana: CPI 11093, Mount Wokomung, Cuyuni-Mazaruni, Guyana.

*Stefania riveroi* (n = 24): MHNLS 10413 (holotype), MHNLS 10414–10416 (paratypes), MHNLS 11160 (paratype), IRSNB 15703, IRSNB 15715–15726, IRSNB 15727 (including µCT scans), IRSNB 15728–15729, IRSNB 15730 (including µCT scans), IRSNB 15740–15741, summit of Yuruaní-tepui, Bolívar State, Venezuela.

***Stefania evansi* clade**

*Stefania evansi* (n = 1): IRSNB 16738 (including µCT scans), Pakatau Creek, Potaro-Siparuni, Guyana.

*Stefania scalae* (n = 6): IRSNB 15674, IRSNB 15693, IRSNB 15694 (including µCT scans), La Escalera, Bolívar State, Venezuela; IRSNB 16724, El Danto, Bolívar State, Venezuela; CPI 10933 and CPI 10943 (including µCT scans), Kamarang Great Falls, Cuyuni-Mazaruni, Guyana.

***Stefania woodleyi* clade**

*Stefania woodleyi* (n = 3): IRSNB 13799, IRSNB 13800 (including µCT scans), IRSNB 13805 (including µCT scans), Kaieteur National Park, Potaro-Siparuni, Guyana.

*Stefania roraimae* (n = 5): IRSNB 15872, IRSNB 15873–15874 (including µCT scans), IRSNB 15875, IRSNB 15876 (including µCT scans), slopes of Maringma-tepui, Cuyuni-Mazaruni, Guyana.

***Stefania ginesi* clade**

*Stefania ginesi* (n = 2): IRSNB 16736–16737 (including µCT scans), summit of Chimantá-tepui, Bolívar State, Venezuela.

*Stefania satelles* (n = 3): MHNLS 10433 (holotype), IRSNB 16728 (including µCT scans), IRSNB 16729, summit of Aprada-tepui, Bolívar State, Venezuela.

**Incertae sedis**

*Stefania breweri* (n = 1): MBUCV 6574 (holotype), summit of Cerro Autana, Amazonas State, Venezuela.

*Stefania goini* (n = 1): AMNH 23193 (holotype), Mount Duida, Amazonas State, Venezuela.

*Stefania oculosa* (n = 1): MHNLS 12961 (holotype), Jaua-tepui, Bolívar State, Venezuela.

*Stefania percristata* (n = 1): MHNLS 12952 (holotype), Jaua-tepui, Bolívar State, Venezuela.

*Stefania schuberti* (n = 3): MHNLS 12917 (holotype), IRSNB 16732 (including µCT scans), IRSNB 16733, Auyán-tepui, Bolívar State, Venezuela.

*Stefania tamacuarina* (n = 2): AMNH 131428 (holotype), MBUCV 6448 (paratype), Pico Tamacuari, Amazonas State, Venezuela.
